# Phylogenetic Analysis of Two New Mitochondrial Genomes of *Singapora shinshana* and *Seriana bacilla* from the Karst Region of Southwest China

**DOI:** 10.3390/genes14071318

**Published:** 2023-06-23

**Authors:** Ni Zhang, Tianyi Pu, Jinqiu Wang, Weiwen Tan, Zhouwei Yuan, Can Li, Yuehua Song

**Affiliations:** 1 School of Karst Science, Guizhou Normal University, Guiyang 550001, China; 2 State Engineering Technology Institute for Karst Desertification Control, Guiyang 550001, China; 3 Guizhou Provincial Key Laboratory for Rare Animal and Economic Insect of the Mountainous Region, Guiyang University, Guiyang 550005, China

**Keywords:** *S. shinshana*, *S. bacilla*, mitogenome, phylogenetic analysis, Karst

## Abstract

Leafhoppers have been identified as a serious threat to different plants. To explore the characteristics of mitogenomes and reveal the phylogenetic positions of two species in the Typhlocybinae, complete mitogenomes of *Singapora shinshana* and *Seriana bacilla* were sequenced and annotated for the first time with lengths of 15,402 bp and 15,383 bp, respectively. The two mitogenomes contained 13 PCGs, 22 tRNA genes and 2 rRNA genes. The genome content, gene order, nucleotide composition, codon usage and amino acid composition are similar to those of other typical mitogenomes of Typhlocybinae. All 13 PCGs started with ATN codons, except for *atp8* (TTA) and *nad5* (TTG). All tRNAs were folded into a typical cloverleaf secondary structure, except for tRNA*-Ser1* and tRNA*-Val*. Moreover, phylogenetic trees were constructed and analyzed based on all the PCGs from 42 mitogenomes using maximum likelihood (ML) and Bayesian inference (BI) methods. The results supported that eleven subfamilies are all monophyletic groups, *S. shinshana* and *S. bacilla* are members of Erythroneurini, but *S. shinshana* and the genus *Empoascanara* have a very close relationship with ((((*Empoascanara sipra*+ *Empoascanara wengangensis*) + *Empoascanara dwalata*) + *Empoascanara gracilis*) + *S. shinshana*), and *S. bacilla* is closely related to the genus *Mitjaevia* ((*Mitjaevia dworakowskae* + *Mitjaevia shibingensis*) + *S. bacilla*). These results provide valuable information for future study of evolutionary relationships in Typhlocybinae.

## 1. Introduction

Phytophagous piercing–sucking insects are insects that feed on the undersides of leaves and insert piercing–sucking mouthparts into the plant tissue to remove plant juices [1]. These insects are some of the main pests that feed on food crops, vegetables and ornamental plants [2]. Cicadellidae, the largest family of Hemiptera, is a type of insect harmful to cereals, vegetables, fruit trees and other trees [3]. *S. shinshana* and *S. bacilla* (Typhlocybinae, Cicadellidae, Hemiptera, Erythroneurini) cause significant damage to leaves of peach trees and potatoes by their sucking behavior, thus leading to yellow-whitish spots on the leaves [4,5]. This damage has a serious impact on the normal growth of agricultural and forestry crops and cause a decline in fruit yield.

The mitochondrial genome plays a crucial part in the phylogenetic and evolutionary analysis of insects. It is widely used to study genome structure and function, population genetic structure and phylogenetic relationships at various taxonomic levels [6,7,8,9,10,11,12,13]. Recently, it has also been used in the phylogenetic discussion of leafhoppers, including Deltocephalinae, Macropsinae, Megophthalminae, Idiocerinae, Iassinae, Coelidiinae, Ledrinae, Evacanthinae, Cicadellinae, Mileewinae and Typhlocybinae [9,14,15,16,17,18,19]. Guizhou is believed to be one of the most well-developed representative karst regions of south China. Many genera and species of Erythroneurini Young, 1952 were discovered in karst areas in Guizhou [20,21,22,23]. In recent years, the majority of studies have focused on the identification and recording of the genera *Singapora* and *Seriana* [24,25,26]. In addition, the seasonal phenology and damage caused by *S. shinshana* have attracted the attention of many experts [4]. However, the lack of mitogenome sequences for *S. shinshana* and *S. bacilla* has limited our understanding of their phylogenetic position within Erythroneurini.

In this study, we successfully sequenced and annotated the complete mitochondrial genome sequences of *S. shinshana* (GenBank accession no. OM048770.1) and *S. bacilla* (OM048922.1) from karst areas for the first time. The newly sequenced mitogenomes were compared with other available mitogenomes of related subfamilies/tribes to (1) analyze the structural and genomic features of the mitogenomes of *S. shinshana* and *S. bacilla*, (2) identify codon usage and (3) construct and elucidate comprehensive phylogenetic relationships (Table 1). The purpose of this work was to enrich the growing mitochondrial database for the Erythroneurini and clarify the phylogenetic position of the two species.

## 2. Materials and Methods

### 2.1. Sample Collection and DNA Extraction

The specimens were collected by a sweeping net in karst areas, and morphological terminology used in this work follows Dietrich (2005) and Song and Li (2013) [46,47]. Detailed collection information is shown in Table 2. The specimens were preserved in 95% ethanol and stored in the insect specimen storage room of Guizhou Normal University. The identified species were selected, and their head, wings and abdomen were removed. Genomic DNA was extracted from thorax muscle tissues and legs by employing a DNeasy Blood & Tissue kit (QIAGEN, Beijing, China). The tissues were ground and incubated at 56 °C for 6 h for complete lysis and total genomic DNA was eluted in 50 µL of double distilled water (ddH_2_O), and the remaining steps were performed according to the manufacturer’s protocol. Genomic DNA was stored at −20 °C.

### 2.2. Mitochondrial Genome Sequencing and Assembly

The complete mitochondrial genomes were sequenced at Berry Genomics (Beijing, China) using an Illumina Novaseq 6000 platform (Illumina, Alameda, CA, USA) with 150 bp paired-end reads. Firstly, the obtained sequence reads were filtered following Zhou et al. [48] and the residual high-quality reads were assembled by an iterative De Bruijin graph de novo assembler, the IDBA-UD toolkit, with a similarity threshold of 98% and *k* values of 40 and 160 bp [49]. The mitogenome was assembled by Geneious Prime 2021 v2021.1.1 using the clean paired reads with default parameters and the mitogenome of *Mitjaevia protuberanta* Song, Li et Xiong, 2011 (Hemiptera: Cicadellidae: Typhlocybinae) (GenBank accession number: NC_047465.1) as the reference [50], and then Geneious Prime software was used to manually map the clean readings to the obtained mitochondrial scaffolds to check the accuracy of the assembly.

### 2.3. Mitogenome Annotation and Sequence Analysis

The assembled mitogenome sequence was subsequently annotated using Geneious Prime and the mitogenome of *M. protuberanta* (GenBank accession number: NC_047465.1) as the reference. All tRNA genes were identified with the MITOS Web Server (http://mitos.bioinf.uni-leipzig.de/index.py, accessed on 12 December 2021) [51]. The annotated mitogenome sequences of *S. shinshana* and *S. bacilla* were deposited in GenBank under the accession numbers OM048770.1 and OM048922.1. The typical secondary structures for tRNAs were drawn with Adobe Illustrator 2021 in accordance with the MITOS predictions. The circular mitogenomic maps were visualized with the CGView server (http://stothard.afns.ualberta.ca/cgview_server/, accessed on 12 December 2021). The base composition, codon usage and relative synonymous codon usage (RSCU) of all protein-coding genes (PCGs) were calculated using MEGA 7.0 [52]. Strand asymmetry was calculated using the following formulae: AT skew = (A − T)/(A + T) and GC-skew = (G − C)/(G + C) [53].

### 2.4. Phylogenetic Analyses

Forty species of Cicadellidae and two outgroups available on GenBank were selected to construct the phylogenetic tree (Table 1). Phylogenetic analyses were performed using the thirteen PCGs of the two species and other leafhopper species. Each PCG was aligned using MAFFT to perform protein alignment [54,55]. Gblocks version 0.91b was used to remove gaps and ambiguously aligned sites [52]. For phylogenetic analyses, the 13 PCG dataset was used to construct phylogenetic trees based on maximum likelihood (ML) and Bayesian inference (BI) using RaxML 8.0.2 [56] and MrBayes 3.2.6 [57], respectively. ML analysis was performed with 1000 rapid bootstrapping replicates using iqtree, and GTR+I+G was considered the best model. BI analysis was performed under the GTR+I+G nucleotide substitution model in MrBayes 3.2.7a with 4 chains and sampling of the chains every 1000 generations [58]. Then, 2 independent runs of 1,000,000 generations were applied.

## 3. Results and Discussion

### 3.1. Genome Organization and Composition

**Figure 1:**
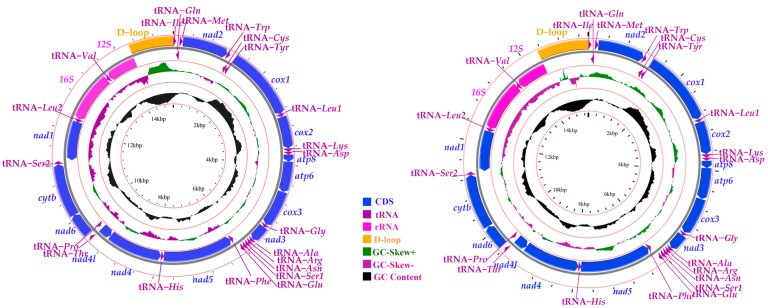
Circular map of the mitochondrial genomes of *S. shinshana* and *S. bacilla*.

As with the reports of most leafhoppers, genome organization and composition were relatively conservative [8]. The complete mitogenomes of *S. shinshana* and *S. bacilla* are double-stranded plasmids with lengths of 15,204 bp and 15,383 bp (Figure 1), which contain 13 PCGs, 22 tRNA genes, 2 rRNA genes, and a control region (Table 3). However, the sequence lengths of *S. shinshana* and *S. bacilla* are different from of those of leafhoppers in complete mitochondrial genomes, based on the length of intergenic space and A+T-rich regions. There was no difference in the mitochondrial genome between the two species, and the content of A and T is higher than G and C. Twenty-three genes are located in the majority strand (J), whereas fourteen genes are encoded in the minority strand (N). Among them, the shortest intergenic space (1 bp) is located between tRNA-*Ser2* and *nad1*, and the longest intergenic space (5 bp) is located between tRNA-*Cys* and tRNA-*Tyr* in *S. shinshana*. However, the shortest intergenic space sequence (1 bp) is located between *atp6* and *cox3*, and the longest intergenic space sequence (11 bp) is located between tRNA-*Gln* and tRNA-*Met* in *S. bacilla*. Additionally, 13 PCG genes were found to overlap by a total of 37 bp and 35 bp in *S. shinshana* and *S. bacilla,* respectively (Table 3). The conserved 8 bp overlapping nucleotide sequence in the two species, located in tRNA-*Typ* and tRNA-*Cys*, is extremely common in Cicadellidae [6,18,42,59], whereas the other 8 bp overlapping nucleotide sequence between *nad6* and *cytb* from *S. shinshana* and the 10 bp overlapping nucleotide sequence from *S. bacilla* between tRNA-*Ser2* and *nad1* could be an accident.

The total nucleotide content per base of *S. shinshana* and *S. bacilla* mitogenomes were, respectively, A (42.9%, 40.8%), T (36.1%, 35.3), G (9.4%, 10.8%) and C (11.7%, 13.0%). Like most insects [60], they had a strong A+T bias, and the content of A+T (78.9%, 76.1%) was significantly higher than that of G+C (21.1%, 23.9%) (Table 4). The overall positive AT skews (0.09, 0.07) and negative GC skews (−0.11, −0.09) indicated that A nucleotide content was higher than T content, and C content was higher than G nucleotide content. The AT skew and GC skew values of the two species are consistent with those reported earlier in leafhoppers [17].

### 3.2. Protein-Coding Genes and Codon Usage

**Figure 2:**
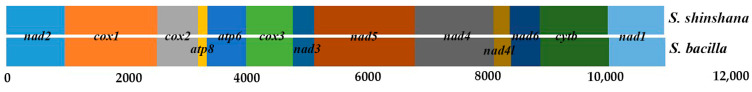
Occurrence and sequence of all the PCGs from *S. shinshana* and *S. bacilla*.

The total lengths of all the PCGs of *S. shinshana* and *S. bacilla* were 11,400 bp and 10,973 bp, accounting for 72.0% and 71.3% of the entire mitogenome, respectively. The order of the PCGs is shown in Figure 2. The longest PCG is *nad5* with 1674 bp and 1675 bp, respectively, and *atp8* is the shortest PCG with 153 bp. This phenomenon also occurs in other species of Cicadellidae [18]. Twelve PCGs of the *S. shinshana* and *S. bacilla* mitogenomes were initiated with the start codon ATN (ATG, ATA, ATT), except *atp8* (TTG). TAA is the most frequent stop codon, but in *cox2* and *nad1* in *S. shinshana* and *cox2* and *nad5* in *S. bacilla*, a single T is used as an incomplete stop codon, which is converted into a complete TAA codon through adding of a polyadenylated tail at the 3′ end [55]. Only four genes, including *nad5*, *nad4*, *nad4L* and *nad1*, are located on the N-strand, and the remaining genes, including *cox1*, *cox2*, *cox3*, *atp8*, *atp6*, *nad2*, *nad3*, *nad6* and *cytb*, are located on the J-strand.

**Figure 3:**
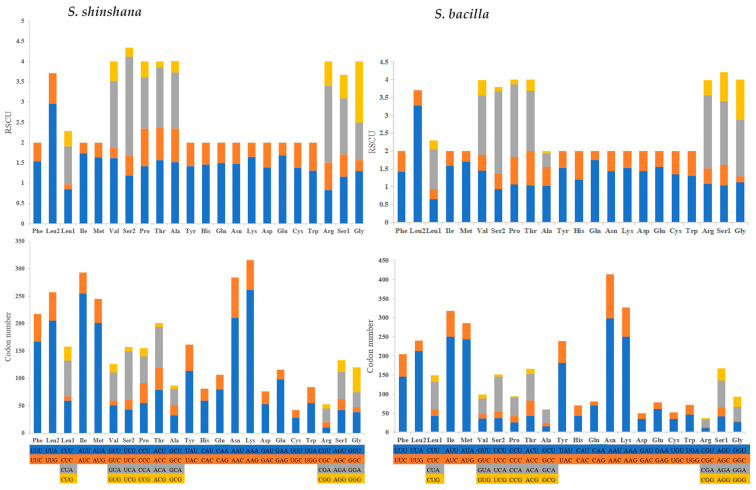
Relative synonymous codon usage (RSCU) and codon number of *S. shinshana* and *S. bacilla*.

The codon usage analysis of *S. shinshana* showed that the most frequently used codon was AAU-Asn (298), followed by AAA-Lys (250), AUU-Ile (250), AUA-Met (243) and UUA-Leu2 (212). However, UUA-Leu2 had the highest RSCU value (3.27) (Table 4 and Figure 3). In a previous study, the most frequently used codon was AUA, and this result is inconsistent with a previous study for Cicadellidae [18]. However, with regard to the codon usage analysis of *S. bacilla*, the most frequently used codons decreased in the following order: AAA-Lys (261), AUU-Ile (255), AAU-Asn (210), UUA-Leu2 (205) and AUA-Met (201) (Table 5). Codon usage analysis revealed that adenine and cytosine are usually located at the third codon. The most frequently used codons end with A or U, therefore, the content of AT is higher than that of GC in PCGs. More generally, this contributes to the AT content of the whole mitogenome.

### 3.3. Ribosomal and Transfer RNA Genes

**Figure 4:**
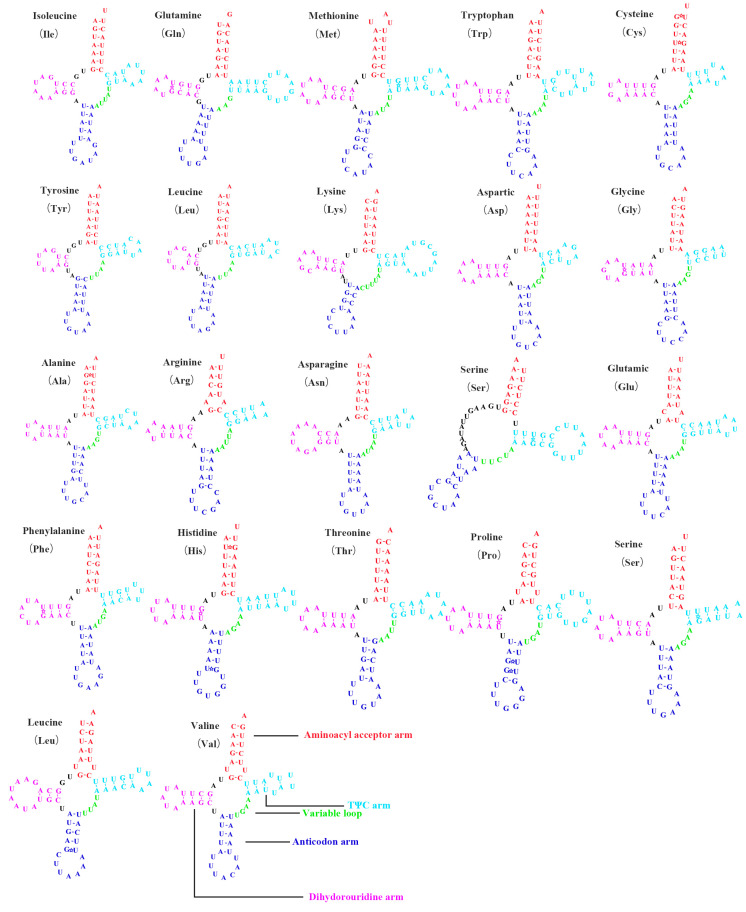

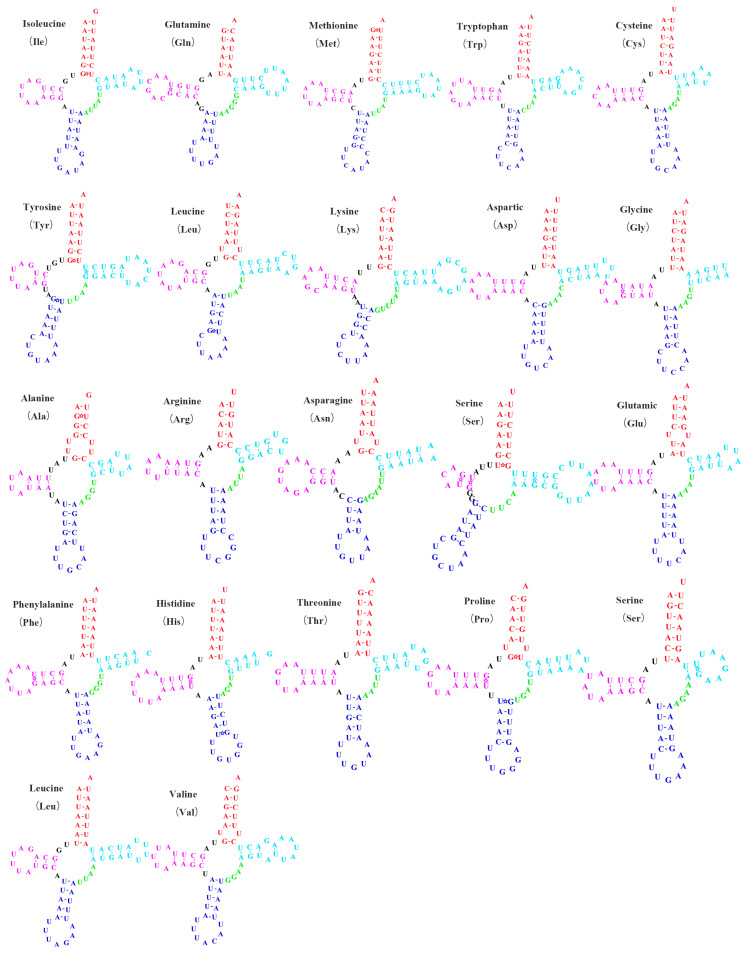
Predicted secondary structure of tRNA genes in the *S. shinshana* and *S. bacilla* mitogenomes.

The *16S rRNA* gene contained 1186 bp and 1185 bp located between tRNA-*Leu2* and tRNA-*Val* in *S. shinshana* and *S. bacilla*, and the *12S rRNA* gene comprised 736 bp and 731 bp situated between tRNA-*Val* and the D-loop in *S. shinshana* and *S. bacilla*. The A+T contents of *16S rRNA* and *12S rRNA* were 83.4%, 85.4% and 81.4%, 80.0%, respectively. Twenty-two tRNA genes from the two species ranged from 61 and 71 bp in both species. Among the 22 tRNA genes, 14 genes are located on the J-strand and 8 genes on the N-strand. This arrangement of tRNA-*Ile* and tRNA-*Gln* is common in Cicadellidae, but a few rearrangements of tRNA-*Ile* and tRNA-*Gln* have been observed in the mitogenome of other subfamilies of Cicadellidae [6,8]. In insects, most tRNA genes are folded into the typical cloverleaf secondary structure, however, tRNA-*Ser1* lacks a dihydrouridine (DHU) arm [61] (Figure 4). Usually, the canonical Watson–Crick base pairings (A–U and C–G) are observed in the tRNA genes, but 16 and 20 noncanonical base pairings (G–U) are found in the DHU arms. The A+T contents in tRNA of *S. shinshana* and *S. bacilla* are 79.3% and 77.0% with a positive AT skew (0.06, 0.02) and a negative GC skew (−0.07, −0.04) (Table 4).

### 3.4. A+T-Rich Region

The mitochondrial A+T-rich region plays an important role in the initiation and regulation of insect replication and transcription in insects [62]. The A+T-rich region is located between *12S rRNA* and tRNA-*Ile* in the mitogenome with a total length of 926 bp and 1045 bp (Table 3) in *S. shinshana* and *S. bacilla*, respectively. The A+T content is 96.7% with both a negative AT skew (−0.24, −0.25) and a GC skew (0.48, 0.47) (Table 4).

### 3.5. Phylogenetic Relationship

The two phylogenetic relationships were constructed using the 13 PCGs by BI and ML methods and then they were merged into one phylogenetic tree. The results indicated that eleven subfamilies are all monophyletic groups, which is consistent with previous reports on phylogenetic research [18,43]. The main topology was as follows: (((Ledrinae + (Evacanthinae + Mileewinae) + (Typhlocybinae + Cicadellinae) + ((Hylicinae + (Coelidiinae +Iassinae)) + (Macropsnae + Megophthalmina))) + Deltocephalina). This result is consistent with phylogenetic relationships of previous studies [18], but different from reports by other researchers, whose results indicate phylogenetic relationships as follows: (Cicadellinae + (Deltocephalinae + (Typhlocybinae + Eurymelinae))) [63] and (Deltocephalinae + ((Cicadellinae + Typhlocybinae) + (Coelidiinae + (Eurymelinae + Megophthalminae + (Smiliinae + (Darthulinae + Centrotinae)))))) [64].

**Figure 5:**
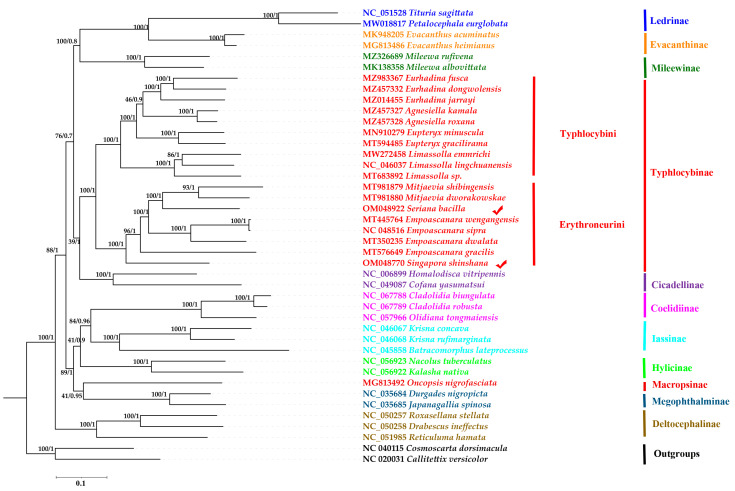
Phylogenetic trees of forty species of Cicadellidae and two groups inferred by maximum likelihood (ML) and Bayesian (BI) methods based on protein-coding genes. The red tick represents the species in this study.

The monophyly of two tribes (Typhlocybini and Erythroneurini) was generally well supported in the subfamily Typhlocybinae, which is consistent with the findings of some previous molecular phylogenetic studies [65,66]. Ten species of Typhlocybini and eight species of Erythroneurini are clustered together, respectively, and most phylogenetic relationships demonstrated a higher nodal support in both ML (BS = 100) and BI (PP = 1) analyses. Our results further confirmed that *S. shinshana* is closely related to the genus *Empoascanara*, while *S. bacilla* and the genus *Mitjaevia* have a closer relationship (Figure 5). In terms of morphological identification, it is easier to distinguish *S. shinshana* from other leafhoppers by observing the appearance. Its body is yellow or yellow-green, but the genera *Seriana*, *Mitjaevia* and *Empoascanara* are difficult to distinguish. Thus, dissection of the male genitals is necessary. Combined with the appearance and the shape of genitalia, *S. shinshana* and *S. bacilla* belong in Erythroneurini (Hemiptera: Cicadellidae: Typhlocybinae). Although the two species belong to the group of Erythronrurini, other species were used to analyze the phylogenetic relationship at the mitochondrial DNA sequence level in order to elucidate their phylogenetic status and verify consistency with traditional taxonomy.

## 4. Conclusions

Consistent with previous results for other Typhlocybinae species, this study presents the mitogenome sequences of *S. shinshana* and *S. bacilla*, which are highly conserved in gene size and organization, highly A+T-biased base composition, codon usage of protein-coding genes and secondary structures of tRNAs. In addition, there are no gene rearrangements. This work provides the basic information to perform comparative analyses and discussion of the mitogenome evolution of Erythroneurini. Phylogenetic analyses support all subfamilies as monophyletic groups and *S. shinshana* and *S. bacilla* as part of Erythroneurini. However, larger scale studies with more taxa are still needed to enrich the mitochondrial genome database and construct more comprehensive phylogenies to support the results of our study. Our study offers valuable data and an efficient framework for the future phylogenetic research of Typhlocybinae.

## Figures and Tables

**Table 1:** List of sequences used to construct the phylogenetic tree.

Subfamily	Species	Accession Number	Reference
Ledrinae	*Tituria sagittata*	NC_051528.1	[27]
	*Petalocephala eurglobata*	MW018817.1	[28]
Evacanthinae	*Evacanthus acuminatus*	MK948205.1	[29]
	*Evacanthus heimianus*	MG813486.1	[30]
Mileewinae	*Mileewa rufivena*	MZ326689.1	[31]
	*Mileewa albovittata*	MK138358.1	[32]
Typhlocybinae	*Eurhadina fusca*	MZ983367.1	Direct submission
	*Eurhadina dongwolensis*	MZ457332.1	Direct submission
	*Eurhadina jarrayi*	MZ014455.1	[33]
	*Agnesiella roxana*	MZ457328.1	Direct submission
	*Agnesiella kamala*	MZ457327.1	Direct submission
	*Eupteryx minuscula*	MN910279.1	[34]
	*Eupteryx gracilirama*	MT594485.1	[35]
	*Limassolla emmrichi*	MW272458.1	[19]
	*Limassolla lingchuanensis*	NC_046037.1	[36]
	*Limassolla* sp.	MT683892.1	[37]
	*Mitjaevia shibingensis*	MT981879.1	[16]
	*Mitjaevia dworakowskae*	MT981880.1	[16]
	*Empoascanara wengangensis*	MT445764.1	[17]
	*Empoascanara sipra*	NC_048516.1	[38]
	*Empoascanara dwalata*	MT350235.1	[39]
	*Empoascanara gracilis*	MT576649.1	[17]
	*Singapora bacilla*	OM048922.1	This study
	*Seriana shinshana*	OM048770.1	This study
Cicadellinae	*Homalodisca vitripennis*	NC_006899.1	Direct submission
	*Cofana yasumatsui*	NC_049087.1	[40]
Colidiinae	*Cladolidia biungulata*	NC_067788.1	[18]
	*Cladolidia robusta*	NC_067789.1	[18]
	*Olidiana tongmaiensis*	NC_057966.1	[18]
Iassinae	*Krisna concava*	NC_046067.1	[8]
	*Krisna rufimarginata*	NC_046068.1	[8]
	*Batracomorphus lateprocessus*	NC_045858.1	[8]
Hylicinae	*Nacolus tuberculatus*	NC_056923.1	[41]
	*Kalasha nativa*	NC_056922.1	[41]
Macropsinae	*Oncopsis nigrofasciata*	MG813492.1	[42]
Megophtthalinae	*Durgades nigropicta*	NC_035684.1	[6]
	*Japanagallia spinosa*	NC_035685.1	[6]
Deltocephalinae	*Roxasellana stellata*	NC_050257.1	[15]
	*Drabescus ineffectus*	NC_050258.1	[15]
	*Reticuluma hamata*	NC_051985.1	[43]
Outgroups (Cercopidae)	*Cosmoscarta dorsimacula*	NC_040115.1	[44]
	*Callitettix versicolor*	NC_020031.1	[45]

**Table 2:** The voucher information of the specimens used for mitochondrial genome sequencing in this study.

Species	Accession Number	Location	Collection Date
*S. shinshana*	GZNU-ELS-20190801	Huajiang, Guizhou (25°42′30″ N, 105°43′20″ E)	10 June 2019
*S. bacilla*	GZNU-ELS-20190601	Shibing, Guizhou (27°0′31.47″ N, 108°01′50.66″ E)	4 August 2019

**Table 3:** Annotations of the mitogenomes of *S. shinshana* (SS) and *S. bacilla* (SB).

Gene	Position		Length		Intergenic		Start Codon		Stop Codon		Strand
	SS	SB	SS	SB	SS	SB	SS	SB	SS	SB	
tRNA*-Ile*	1–63	1–64	63	64	0	0					J
tRNA*-Gln*	66–133	63–129	68	67	2	−2					N
tRNA*-Met*	134–202	141–209	69	69	0	11					J
*nad2*	203–1174	210–1181	972	972	0	0	ATT	ATA	TAA	TAA	J
tRNA*-Trp*	1173–1240	1180–1248	68	69	−2	−2					J
tRNA*-Cys*	1233–1293	1241–1301	61	61	−8	−8					N
tRNA*-Tyr*	1299–1361	1311–1378	63	68	5	9					N
*cox1*	1364–2902	1381–2916	1539	1536	2	2	ATG	ATG	TAA	TAA	J
tRNA*-Leu1*	2898–2964	2920–2985	67	66	−5	3					J
*cox2*	2965–3643	2986–3664	679	679	0	0	ATA	ATT	T	T	J
tRNA*-Lys*	3644–3714	3665–3735	71	71	0	0					J
tRNA*-Asp*	3714–3775	3736–3801	62	66	−1	0					J
*atp8*	3774–3926	3801–3953	153	153	−2	−1	TTG	TTG	TAA	TAA	J
*atp6*	3923–4573	3950–4600	651	651	−4	−4	ATA	ATA	TAA	TAA	J
*cox3*	4574–5353	4602–5381	780	780	0	1	ATG	ATG	TAA	TAA	J
tRNA*-Gly*	5356–5417	5387–5448	62	62	2	5					J
*nad3*	5418–5771	5449–5802	354	354	0	0	ATA	ATT	TAG	TAA	J
tRNA*-Ala*	5770–5832	5805–5865	63	61	−2	2					J
tRNA*-Arg*	5835–5894	5866–5924	60	59	2	0					J
tRNA*-Asn*	5894–5958	5925–5990	65	66	−1	0					J
tRNA*-Ser1*	5958–6024	5990–6057	67	68	−1	−1					J
tRNA*-Glu*	6024–6089	6061–6125	66	65	−1	3					J
tRNA*-Phe*	6090–6153	6124–6188	64	65	0	−2					N
*nad5*	6154–7827	6189–7863	1674	1675	0	0	ATT	ATT	TAA	T	N
tRNA*-His*	7828–7893	7861–7924	66	64	0	−3					N
*nad4*	7894–9201	7925–9247	1308	1323	0	0	ATA	ATA	TAA	TAA	N
*nad4L*	9201–9476	9247–9531	276	285	−1	−1	ATG	ATA	TAA	TAA	N
tRNA*-Thr*	9479–9543	9536–9600	65	65	2	4					J
tRNA*-Pro*	9544–9609	9601–9666	65	66	0	0					N
*nad6*	9612–10,103	9669–10,154	492	486	2	2	ATT	ATT	TAA	TAA	J
*cytb*	10,096–11,232	10,159–11,295	1137	1137	−8	4	ATG	ATG	TAA	TAA	J
tRNA*-Ser2*	11,232–11,295	11,295–11,358	64	64	−1	−1					J
*nad1*	11,297–12,227	11,349–12,290	931	942	1	−10	ATT	ATT	T	TAA	N
tRNA*-Leu2*	12,228–12,292	12,291–12,355	65	65	0	0					N
*16S*	12,293–13,478	12,356–13,540	1186	1185	0	0					N
tRNA*-Val*	13,479–13,542	13,541–13,607	64	67	0	0					N
*12S*	13,543–14,278	13,608–14,338	736	731	0	0					N
D-loop	14,279–15,204	14,339–15,383	926	1045	0	0					J

**Table 4:** Nucleotide compositions, AT skew and GC skew in different regions of *S. shinshana* and *S. bacilla*.

*S. shinshana*	Size (bp)	A%	C%	G%	T%	A+T%	AT Skew	GC Skew
Full genome	15,204	42.9	11.7	9.4	36.1	78.9	0.09	−0.11
PCGs	10,946	42.7	12.8	10.5	34.0	76.7	0.11	−0.53
1st codon position	3649	45.9	11.8	10.4	32.0	77.9	0.18	−0.06
2nd codon position	3649	40.1	12.9	11.5	35.0	75.1	0.07	−0.06
3rd codon position	3648	42.1	13.7	9.6	35	77.1	0.09	−0.18
tRNA	1436	42.0	11.1	9.6	37.3	79.3	0.06	−0.07
rRNA	1922	47.6	10.8	6.6	35.1	82.7	0.15	−0.24
D-loop	926	36.5	0.9	2.5	60.2	96.7	−0.24	0.48
S. bacilla								
Full genome	15,383	40.8	13.0	10.8	35.3	76.1	0.07	−0.09
PCGs	10,973	39.4	14.5	12.4	33.7	73.1	0.08	−0.08
1st codon position	3658	40.3	15.1	14.4	30.0	70.3	0.15	−0.02
2nd codon position	3658	36.3	16.4	11.8	35.0	71.3	0.02	−0.16
3rd codon position	3657	41.5	11.8	11.2	36	77.5	0.07	−0.03
tRNA	1438	39.2	12.0	11.0	37.8	77.0	0.02	−0.04
rRNA	1916	48.1	11.7	6.5	33.7	81.8	0.18	−0.29
D-loop	926	36.5	0.9	2.5	60.2	96.7	−0.25	0.47

**Table 5:** Codon and relative synonymous codon usage (RSCU) of 13 PCGs of *S. shinshana* and *S. bacilla*.

Amino Acid	Codon	Count/RSCU				Amino Acid	Codon	Count/RSCU			
		*S. shinshana*		*S. bacilla*				*S. shinshana*		*S. bacilla*	
Phe	UUU	145	1.42	167	1.54	Tyr	UAU	181	1.52	114	1.42
	UUC	59	0.58	50	0.46		UAC	57	0.48	47	0.58
Leu2	UUA	212	3.27	205	2.96	His	CAU	42	1.2	59	1.46
	UUG	28	0.43	52	0.75		CAC	28	0.8	22	0.54
Leu1	CUU	42	0.65	59	0.85	Gln	CAA	70	1.75	80	1.5
	CUC	18	0.28	8	0.12		CAG	10	0.25	27	0.5
	CUA	72	1.11	65	0.94	Asn	AAU	298	1.44	210	1.48
	CUG	17	0.26	26	0.38		AAC	116	0.56	74	0.52
Ile	AUU	250	1.58	255	1.74	Lys	AAA	250	1.53	261	1.65
	AUC	67	0.42	38	0.26		AAG	76	0.47	55	0.35
Met	AUA	243	1.71	201	1.64	Asp	GAU	35	1.43	53	1.39
	AUG	42	0.29	44	0.36		GAC	14	0.57	23	0.61
Val	GUU	36	1.45	51	1.62	Gln	GAA	61	1.56	98	1.69
	GUC	11	0.44	8	0.25		GAG	17	0.44	18	0.31
	GUA	41	1.66	52	1.65	Cys	UGU	35	1.35	29	1.38
	GUG	11	0.44	15	0.48		UGC	17	0.65	13	0.62
Ser2	UCU	37	0.93	43	1.19	Trp	UGA	46	1.3	55	1.31
	UCC	17	0.43	18	0.5		UGG	25	0.7	29	0.69
	UCA	92	2.31	88	2.43	Arg	CGU	10	1.08	11	0.83
	UCG	5	0.13	8	0.22		CGC	4	0.43	9	0.68
Pro	CCU	25	1.06	55	1.42		CGA	19	2.05	25	1.89
	CCC	18	0.77	36	0.93		CGG	4	0.43	8	0.6
	CCA	48	2.04	49	1.26	Ser1	AGU	41	1.03	42	1.16
	CCG	3	0.13	15	0.39		AGC	23	0.58	20	0.55
Thr	ACU	43	1.04	79	1.57		AGA	71	1.79	50	1.38
	ACC	40	0.96	40	0.8		AGG	32	0.81	21	0.58
	ACA	70	1.69	75	1.49	Gly	GGU	26	1.12	39	1.3
	ACG	13	0.31	7	0.14		GGC	4	0.17	8	0.27
Ala	GCU	15	1.02	33	1.52		GGA	37	1.59	28	0.93
	GCC	8	0.54	18	0.83		GGG	26	1.12	45	1.5
	GCA	35	0.37	30	1.38	*	UAA	168	1.61	155	1.38
	GCG	1	0.07	6	0.28		UAG	41	0.39	33	0.62

* stands for stop codon.

## Data Availability

The complete mitochondrial genome sequences of *S. shinshana* and *S. bacilla* are available at GenBank OM048922.1 and OM048770.1.
